# Supplementary material for: Evaluation of novel glucose-related blood biomarkers for predicting in-hospital mortality in patients with acute ischemic stroke
Source: PLoS One. 2025 May 21;20(5):e0324291. doi: 10.1371/journal.pone.0324291 (PMC12094732; doi:10.1371/journal.pone.0324291)
Supplement: Attachment — Submitted filename: Responses to Reviewers_04_21_2025_R2_Final.pdf [file pone.0324291.s004.pdf]

Point-by-point Reply

Review Comments to the Author

Reviewer #1:

Dear Author
The revised manuscript is addressed properly with reviewer comments. So now the manuscript is ready to accept and publish

Response: We warmly thank the reviewer for the thoughtful review and kind remarks regarding our work.

Reviewer #2:

Thank you for providing the revised comments. The bias inherent to the study are mostly transparent and the methods are now clearer. I agree with the revised conclusion The lack of including diabetes, a strong confounder, in the multivariate analysis needs to be mentioned explicitly in the limitations section.

Response: We truly appreciate the reviewer's insightful comments. The manuscript has been revised accordingly, and the suggested sentences have been added in the discussion at Line 414-415.

Journal requirements:

Please review your reference list to ensure that it is complete and correct. If you have cited papers that have been retracted, please include the rationale for doing so in the manuscript text, or remove these references and replace them with relevant current references. Any changes to the reference list should be mentioned in the rebuttal letter that accompanies your revised manuscript. If you need to cite a retracted article, indicate the article's retracted status in the References list and also include a citation and full reference for the retraction notice.

Response: We have verified all 39 references to ensure that none of them have been retracted.
